# Nuclear receptor binding protein 1 correlates with better prognosis and induces caspase-dependent intrinsic apoptosis through the JNK signalling pathway in colorectal cancer

**DOI:** 10.1038/s41419-018-0402-7

**Published:** 2018-03-22

**Authors:** Yi Liao, Zihuan Yang, Jintuan Huang, Hao Chen, Jun Xiang, Senmao Li, Chunyu Chen, Xuan He, Feng Lin, Zuli Yang, Jianping Wang

**Affiliations:** 1 Department of Gastrointestinal Surgery, The Sixth Affiliated Hospital, Sun Yat-sen University, 510655 Guangzhou, People’s Republic of China; 2 Guangdong Provincial Key Laboratory of Colorectal and Pelvic Floor Disease, The Sixth Affiliated Hospital, Sun Yat-sen University, 510655 Guangzhou, People’s Republic of China; 3 Department of Colorectal Surgery, The Sixth Affiliated Hospital, Sun Yat-sen University, 510655 Guangzhou, People’s Republic of China

## Abstract

Nuclear receptor binding protein 1 (NRBP1) is a ubiquitously expressed and highly conserved pseudokinase that has important roles in cellular homoeostasis. Despite recent advances in understanding the biology of NRBP1, the role of NRBP1 and its underlying mechanism in colorectal cancer (CRC) have not been fully elucidated. In the present study, we observed that NRBP1 expression levels were significantly reduced in CRC tissues compared with corresponding adjacent normal tissues, and high NRBP1 expression correlated with better prognosis in CRC. Overexpression of NRBP1 inhibited CRC cell proliferation and promoted apoptosis in vitro and in vivo. In contrast, knockdown of NRBP1 expression increased cell proliferation and decreased the percentage of apoptotic cells. Moreover, overexpression of NRBP1 activated caspase-dependent intrinsic apoptosis. In addition, we further discovered that NRBP1 regulated the apoptotic pathway through interaction with JNK. Finally, NRBP1 overexpression led to attenuated CRC growth in a xenograft mouse model. Our study illustrates the suppressor role of NRBP1 in CRC and provides a potential therapeutic target.

## Introduction

Colorectal cancer (CRC) is a significant health problem. With an estimated 1.4 million cases and 693,900 deaths occurring, CRC continues to be the third most frequently diagnosed cancer in men worldwide and the second in women^1^. In China, CRC has become the fourth most common carcinoma and the fifth most common cause of mortality^2^. The occurrence and development of CRC involves the progressive accumulation of oncogene activation and inactivation of tumour-suppressor genes, such as P53, PTEN, APC and KRAS^3,4^. The alteration of multiple signal pathways caused by mutations in components contribute to the carcinogenesis of CRC^5^. Understanding the molecular mechanism might lay the foundation for cancer prevention, early diagnosis and effective treatment.

Nuclear receptor binding protein 1 (NRBP1) is an adapter protein that is ubiquitously expressed across all cell types. The NRBP1 gene is located on human chromosome 2p23 and is highly conserved between species. NRBP1 has been shown to contain a potential Src homology 2 (SH2) domain-binding region, a kinase-like domain, a myeloid leukaemia factor 1 (MLF1)-binding region, a BC-binding box and a transforming growth factor β1-stimulated clone 22 (TSC22)-binding region^6–8^. Regrettably, except for its interaction with a handful of key transcription factors, knowledge about the function of NRBP1 is limited. NRBP1 has been shown to bind to activated Rac3, MLF1 oncoprotein and JAB1, leading to redistribution of the Golgi marker p58, inhibition of cellular differentiation and inhibition of JAB1-mediated AP-1 activation^6,9,10^. Moreover, NRBP1 has been shown to interact with the elongin BC complex, a key component of the ubiquitination machinery, and the loss of NRBP1 in the intestine results in the accumulation of Sall4, a key mediator of stem cell fate, and of Tsc22d2^11^.

The expression of NRBP1 has been observed in several human cancer lines, including breast cancer cell lines, CRC lines, lung cancer lines and macrophage-like cell lines^7^. Only recently has NRBP1 been proposed to have a role in cancer progression, but the role of NRBP1 is not fully understood because NRBP1 has been reported to have pro- or anti-cancer progression functions. In lung cancer and breast cancer, NRBP1 serves as a potential tumour suppressor^11,12^. In contrast, it has been proposed to exert tumour-promoting effects in prostate cancer^13^. Reduced NRBP1 mRNA expression was detected in colorectal carcinoma^11^, implying that NRBP1 might be a tumour-suppressor gene in CRC. However, the role of NRBP1 in CRC has not been fully elucidated, and whether NRBP1 has a tumour suppressive function in CRC cells needs to be further validated. Therefore, we performed this study to explore the expression, detailed function and underlying mechanism of NRBP1 in CRC.

## Results

### Expression of NRBP1 is downregulated in CRC

**Fig. 1:**
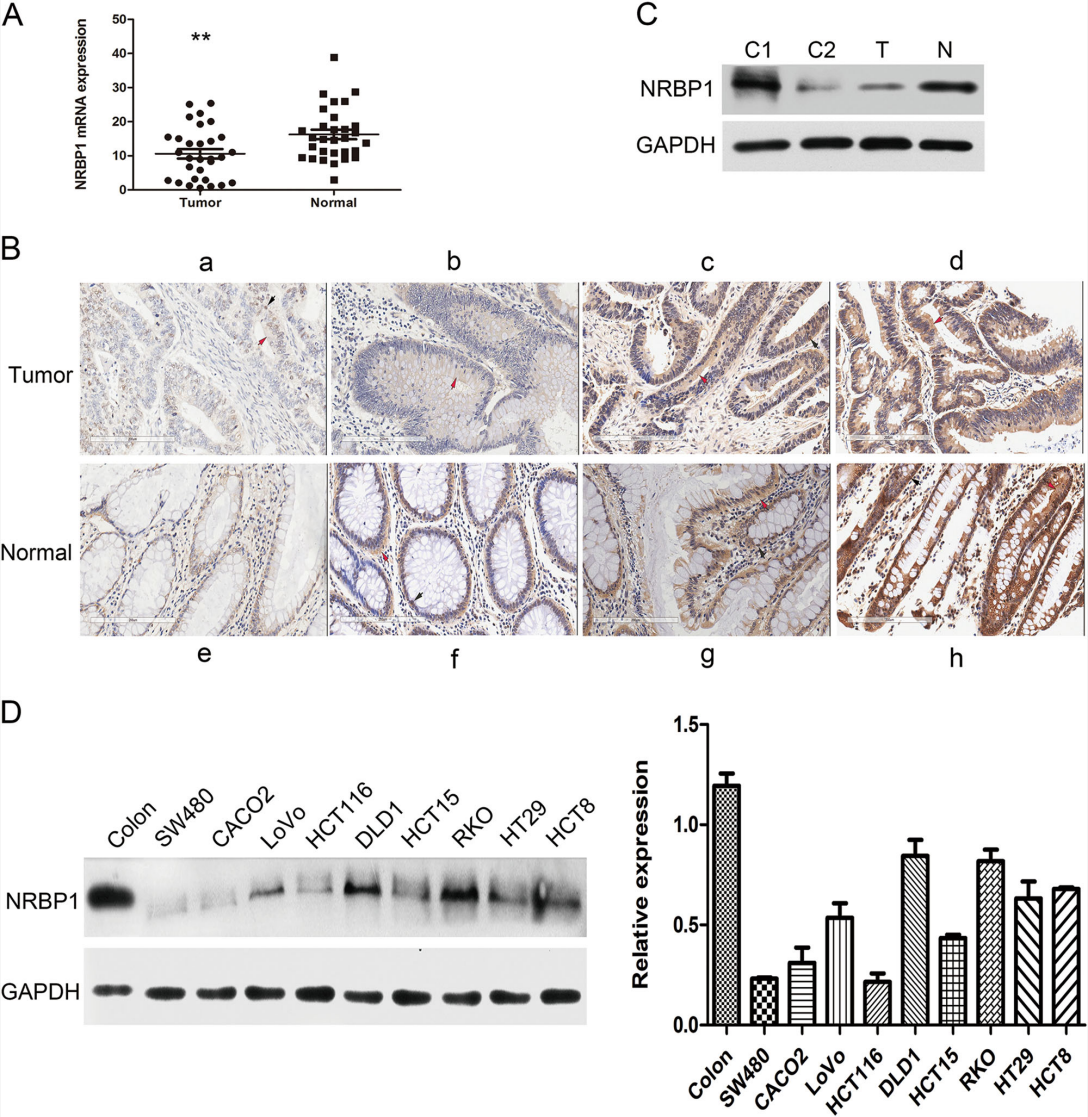
Expression of NRBP1 is downregulated in CRC. **a** qRT-PCR analysis of the expression levels of NRBP1 in 30 pairs of fresh-frozen primary CRC tissues and their matched normal tissues adjacent to cancer tissues. Three experiments were done. NRBP1 mRNA expression was normalised to β-actin mRNA, which used as the internal reference. The data are expressed as the mean ± standard deviation. (**P < 0.01). **b** IHC staining to detect NRBP1. **a** Weak staining of NRBP1 in CRC tissues. **b**–**d** More intense staining of NRBP1 in CRC tissues. **e** Weak staining of NRBP1 in normal colorectal tissues. **f**–**h** More intense staining of NRBP1 in normal colorectal tissues. Red arrow indicates cytoplasm staining. Black arrow indicates nucleus staining. **c** The expression levels of NRBP1 protein were analysed by western blot in CRC tissue and matched normal colorectal tissue. GAPDH was used as loading control. N normal tissue, T tumour tissue, C1 protein from stomach tissue was used as positive control, C2 protein from thymus tissue was used as negative control. **d** Western blot was used to investigate NRBP1 protein expression in nine CRC cell lines and normal colorectal tissue

To examine the expression status of NRBP1 in CRC, RNA was extracted from 30 pairs of fresh-frozen primary CRC tissues and their matched normal tissues adjacent to cancer tissues, and NRBP1 mRNA levels in these samples were measured by quantitative real-time PCR (qRT-PCR). The expression of NRBP1 mRNA was normalised to β-actin mRNA, which served as a control for the input cDNA. Compared with adjacent normal tissues, the expression levels of NRBP1 mRNA were markedly downregulated in cancer tissues (16.3 ± 7.72 vs 10.6 ± 7.63, *P* = 0.008, Fig. 1a). To further validate the NRBP1 expression status, we employed immunohistochemistry (IHC) analysis in another 30 pairs of matched CRC and normal tissues. The NRBP1 protein was found to be localised mainly in the cytoplasm; however, expression of NRBP1 protein was also detected in the nucleus in some samples. The expression of NRBP1 protein tends to be lower in cancer tissues than in normal tissues. (Fig. 1b; Table 1). Negative or low expression of NRBP1 was detected in 50% (15/30) of tumour tissues compared to 13.3% (4/30) of paired normal tissues. High expression was found in 50% (15/30) of tumour tissues compared to 86.7% (26/30) of matched normal tissues. The percentage of tumour tissues highly expressing NRBP1 was much smaller than that in matched normal tissues (*P* = 0.002).

**Table 1:** Comparison of NRBP1 expression in CRC tissue and paired normal tissue by IHC

		NRBP1 expression		
	Cases	Negative or low	High	*P* value
CRC tissue	30	15 (50%)	15 (50%)	0.002
Paired normal tissue	30	4 (13.3%)	26 (86.7%)	

Then, NRBP1 protein expression levels were analysed in CRC and matched noncancerous tissues and in nine human CRC cell lines by western blot. Stomach and thymus tissues were used as the positive and negative controls, respectively. Compared with normal colorectal tissue, a low level of NRBP1 protein was detected in cancer tissue (Fig. 1c), and NRBP1 expression was low in the nine cell lines examined, especially in SW480 and HCT116 cells. The levels of NRBP1 were relatively high in DLD1 and RKO cells in the nine cell lines (Fig. 1d). Therefore, SW480 and HCT116 cells were selected for overexpression experiments, whereas DLD1 and RKO cells were selected for knockdown experiments. Together, these data implicated decreased NRBP1 expression as a characteristic marker for CRC.

### High NRBP1 expression correlates with better prognosis in CRC

**Fig. 2:**
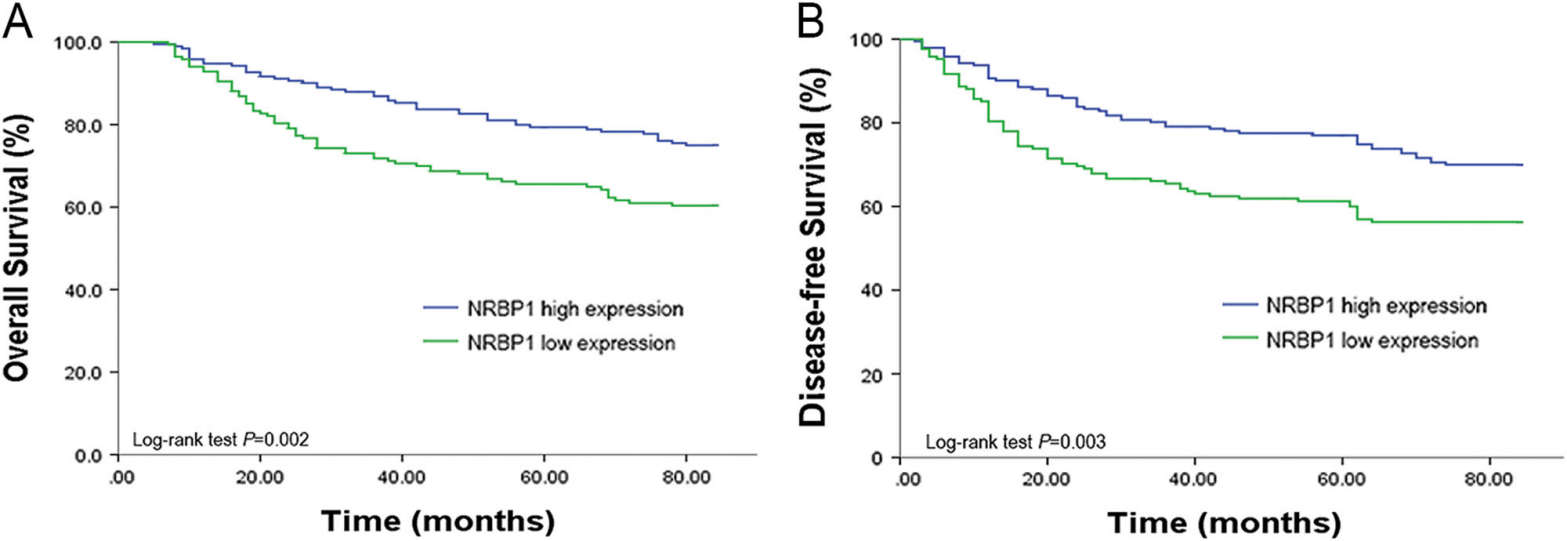
High NRBP1 expression correlate with better prognosis in CRC. Kaplan–Meier curves displaying **a** overall survival and **b** disease-free survival in patients with primary CRC based on NRBP1 expression status (high or low expression). High expression patients had better survival than low expression patients. *P* values were defined by log-rank test

To explore the correlation between NRBP1 expression levels and clinicopathological parameters of CRC, IHC was performed on the tissue microarrays (TMAs), including tissues from 360 primary CRC specimens. Among these samples, a high level of NRBP1 expression was detected in 192 cases (53.3%), and a low level of NRBP1 expression was detected in 168 cases (46.7%). The association between NRBP1 levels and clinicopathological characteristics are summarised in Table 2. Early tumour/node/metastasis (TNM) stage (stage I + II) was frequently identified with high NRBP1 expression (*P* = 0.012). However, we did not observe a significant association between NRBP1 expression and age, gender, depth of infiltration, lymph node metastasis and distant metastasis, tumour differentiation, tumour histological type or location (Table 2). Kaplan–Meier analysis was used to detect any potential correlation between survival and NRBP1 level. Patients with high NRBP1 levels were significantly associated with longer overall survival (OS) and disease-free survival (DFS) than those with low NRBP1 levels (*P* = 0.002 and *P* = 0.003, respectively, Fig. 2a, b). The 5-year OS and DFS of patients with high NRBP1 levels were 79.7 and 78.1%, respectively, whereas it was only 66.1% for 5-year OS and 61.9% for 5-year DFS for patients with low NRBP1 levels.

**Table 2:** Correlation of NRBP1 expression with clinicopathological features in CRC tissues

Characteristcs	Cases	NRBP1 expression		*P* value
		Low	High	
Gender				
Female	149	70	79	0.920
Male	211	98	113	
Age				
≤60	165	84	81	0.138
>60	195	84	111	
Location				
Colon	228	101	127	0.236
Rectum	122	67	65	
Differentiation				
Well/moderate	293	134	159	0.458
Poorly	67	34	33	
Histological type				
Adenocarcinoma	318	150	168	0.572
Mucous carcinoma	42	18	24	
Preoperative CEA level (ng/mL)				
≤5	190	91	99	0.621
>5	170	77	93	
pT (invasion depth)				
T1 + T2	63	29	34	0.911
T3 + T4	297	139	158	
pN (lymph node metastasis)				
N0	214	94	120	0.207
N1–2	146	74	72	
pM (distant metastasis)				
M0	317	142	175	0.053
M1	43	26	17	
TNM stage				
I + II	199	81	118	0.012
III + IV	161	87	74

In addition, a Cox regression model was established to analyse the effect of each variable on OS and DFS. In terms of OS, univariate analysis revealed that tumour NRBP1 expression status, gender, differentiation grade, pT stage, pN stage, pM stage and TNM stage were prognostic factors. The results of the multivariate analysis showed that tumour NRBP1 expression status, gender, differentiation grade, pM stage and TNM stage were independent prognostic factors (Table 3). Regarding DFS, NRBP1 expression status, differentiation grade, pT stage, pN stage, pM stage and TNM stage were identified as prognostic factors. Multivariate analysis indicated that the independent prognostic variables for DFS included NRBP1 expression status, differentiation grade, pM stage and TNM stage (Table 4).

**Table 3:** Univariate and multivariate analysis of overall survival in CRC patients

Variables	Univariate analysis		Multivariate analysis	
	HR (95% CI)	*P*	HR (95% CI)	*P*
Gender				
Female	1	0.031	1	0.037
Male	1.503 (1.037, 2.177)		1.497 (1.025, 2.186)	
Age				
≤60	1	0.747		
>60	1.603 (0.733,1.543)			
Location				
Colon	1	0.651		
Rectum	1.093 (0.743,1.610)			
Differentiation				
Well/moderate	1	<0.001	1	<0.001
Poorly	3.654 (2.482,5.380)		2.699 (1.811, 4.022)	
Histological type				
Adenocarcinoma	1	0.864		
Mucous carcinoma	0.949 (0.521,1.727)			
Preoperative CEA level (ng/mL)				
≤5	1	0.77		
>5	1.057 (0.729, 1.532)			
pT (invasion depth)				
T1 + T2	1	0.009		
T3 + T4	2.299 (1.233, 4.284)			
pN (lymph node metastasis)				
N0	1	<0.001		
N1–2	2.075 (1.430, 3.012)			
pM (distant metastasis)				
M0	1	<0.001	1	<0.001
M1	11.327 (7.301, 17.572)		6.295 (3.881, 10.210)	
TNM stage				
I + II	1	<0.001	1	0.008
III + IV	3.465 (2.334, 5.143)		1.851 (1.179, 2.908)	
NRBP1 expression				
High	1	0.002	1	0.014
Low	1.810 (1.243, 2.635)		1.622 (1.104, 2.384)

**Table 4:** Univariate and multivariate analysis of disease-free survival in CRC patients

Variables	Univariate analysis		Multivariate analysis	
	HR (95% CI)	*P*	HR (95% CI)	*P*
Gender				
Female	1	0.103		
Male	1.333 (0.944, 1.882)			
Age				
≤60	1	0.711		
>60	1.068 (0.755, 1.509)			
Location				
Colon	1	0.194		
Rectum	1.279 (0.883, 1.843)			
Differentiation				
Well/moderate	1	<0.001	1	<0.001
Poorly	3.434 (2.387, 4.941)		2.515 (1.727, 3.662)	
Histological type				
Adenocarcinoma	1	0.925		
Mucous carcinoma	1.026 (0.599, 1.758)			
Preoperative CEA level (ng/mL)				
≤5	1	0.454		
>5	1.140 (0.809, 1.608)			
pT (invasion depth)				
T1 + T2	1	0.018		
T3 + T4	1.918 (1.120,3.286)			
pN (lymph node metastasis)				
N0	1	<0.001		
N1–2	2.043 (1.446, 2.885)			
pM (distant metastasis)				
M0	1	<0.001	1	<0.001
M1	11.605 (7.718, 17.450)	6.859 (4.377, 10.748)		
TNM stage				
I + II	1	<0.001	1	0.004
III + IV	3.482 (2.413, 5.023)		1.856 (1.219, 2.826)	
NRBP1 expression				
High	1	0.003	1	0.043
Low	1.686 (1.192, 2.285)		1.437 (1.011, 2.041)	

### Ectopic expression of NRBP1 inhibits CRC cell proliferation and promotes apoptosis

**Fig. 3:**
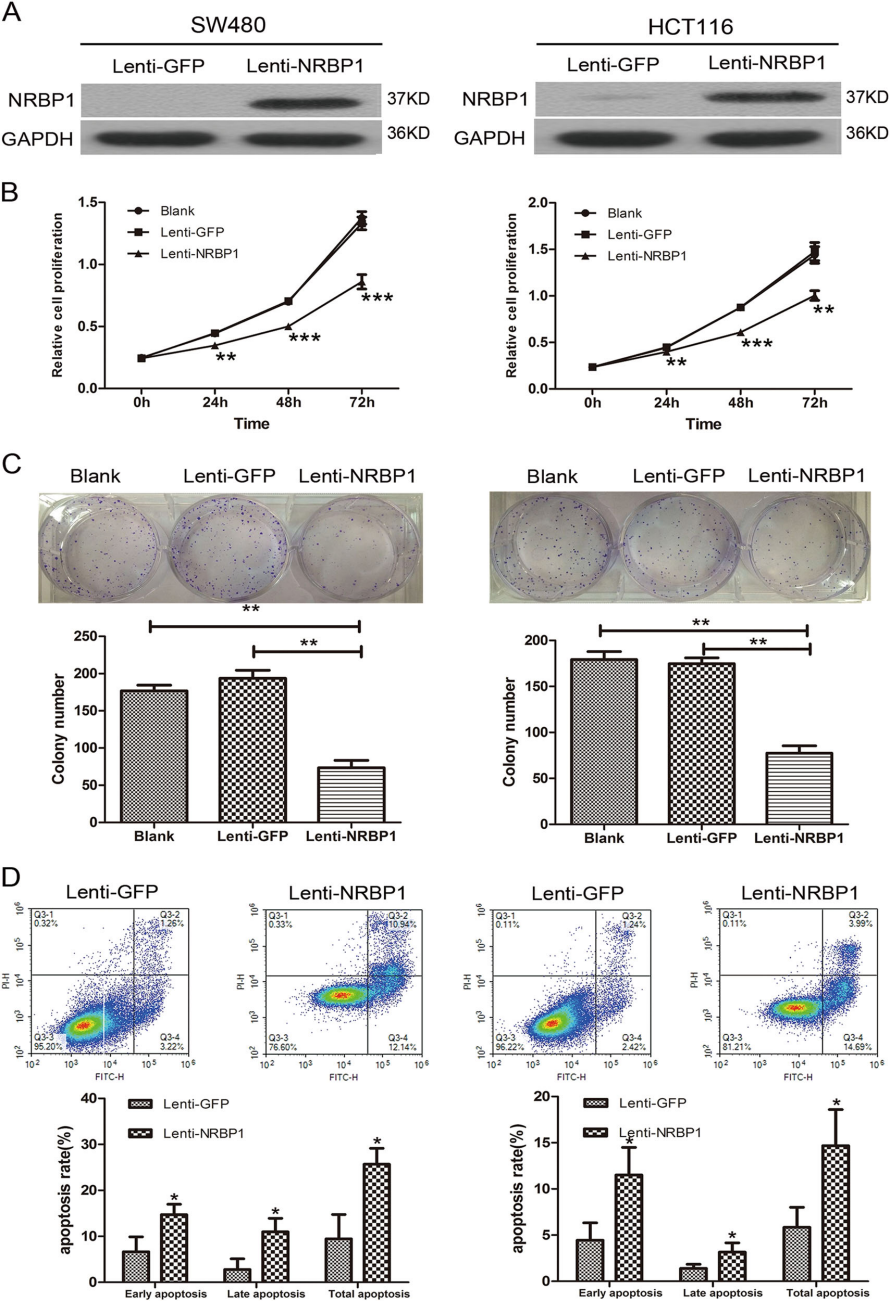
Effects of ectopic expression of NRBP1 on proliferation and apoptosis of SW480 and HCT116 cells. **a** Increase levels of NRBP1 in SW480 and HCT116 cells transduced with lenti-NRBP1 was confirmed by western blot. **b** The short time effect of ectopic NRBP1 on the tumour cell proliferation was evaluated by CCK-8 at 0, 24, 48 and 72 h. **c** Representative pictures (top) of colony formation assay for crystal violet-stained cells and quantified analysis (bottom). Values represent three independent experiments. **d** Cell apoptosis was measured by flow cytometry analysis following Annexin V and PI staining. Experiment was repeated three times in triplicate. Images show a representative result (top). Q3-1 necrosis cells. Q3-2 late apoptosis cells. Q3-3 normal cells. Q3-4 early apoptosis cells. Graphs (bottom) show quantitative analysis of early apoptosis, late apoptosis and total apoptosis (*P < 0.05, **P < 0.01, ***P < 0.001)

We used a lentivirus system to generate stable NRBP1 overexpression CRC lines. Lenti-NRBP1 or lenti-GFP were used to transduce SW480 and HCT116 cells. Western blot analysis showed that NRBP1 protein expression was significantly elevated in SW480 and HCT116 cells transduced with lenti-NRBP1 (Fig. 3a). The CCK-8 and colony formation assays revealed a significant inhibition of cell proliferation in SW480 and HCT116 cells treated with lenti-NRBP1 compared to control-treated cells and blank cells (Fig. 3b, c). To determine whether the growth inhibitory effect NRBP1 exerts on CRC cells is due to enhanced apoptosis, the apoptosis ratios were analysed by double staining the cells with Annexin V and PI using flow cytometry. Annexin V/PI staining indicated that overexpression of NRBP1 increased both early and late apoptosis ratios in both cells (Fig. 3d). NRBP1 increased total apoptotic effects from 9.43 ± 5.32% to 29.69 ± 3.45% in SW480 cells and from 5.84 ± 2.17% to 14.67 ± 3.92% in HCT116 cells.

### Knockdown of NRBP1 promotes CRC cell proliferation and inhibits apoptosis

**Fig. 4:**
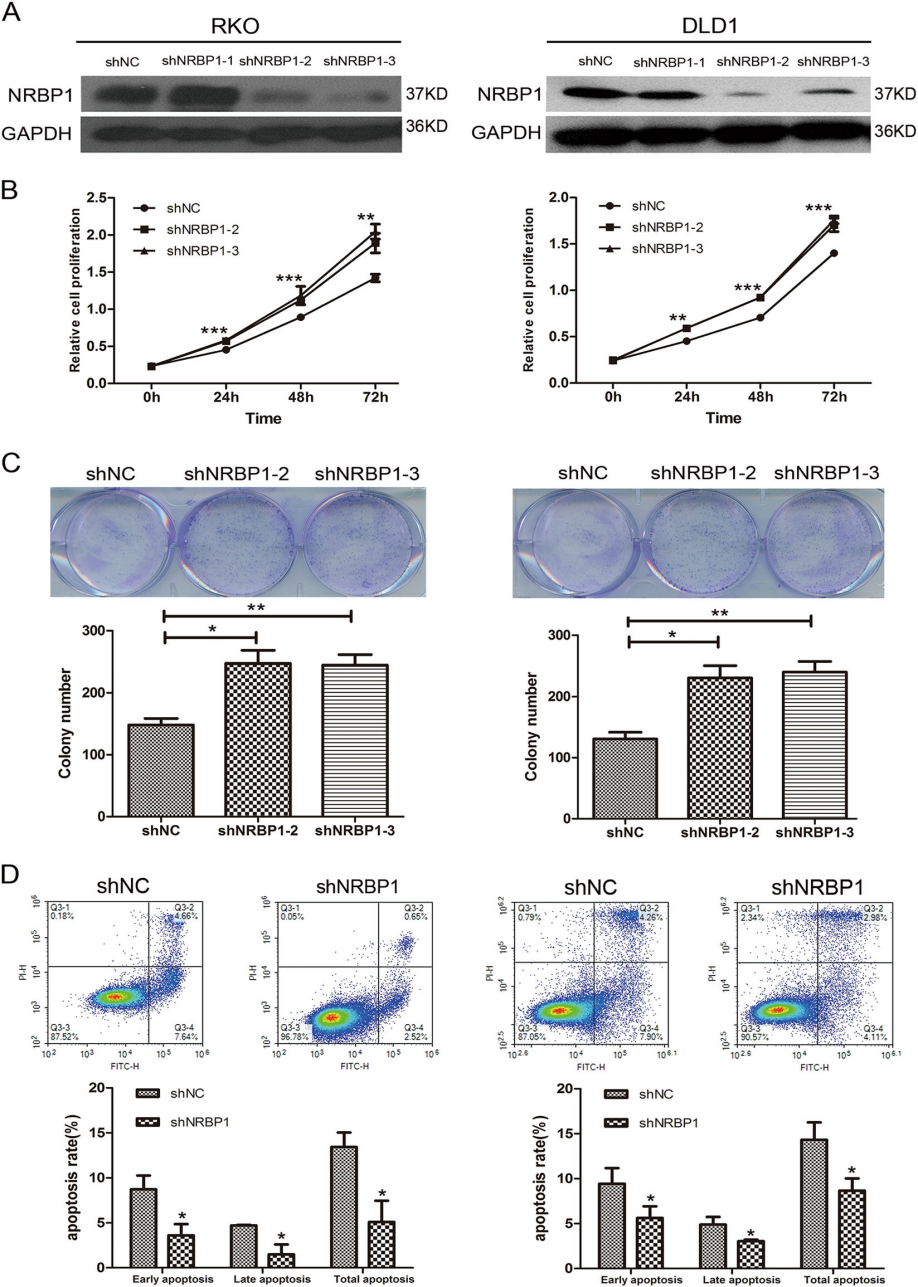
Effects of knockdown of NRBP1 on proliferation and apoptosis of RKO and DLD1 cells. **a** Decreased levels of NRBP1 in RKO and DLD1 cells transduced with sh2 and sh3 was confirmed by western blot. **b** The short time effect of silencing NRBP1 on the tumour cell proliferation was evaluated by CCK-8 at 0, 24, 48 and 72 h. **c** Representative pictures (top) of colony formation assay for crystal violet-stained cells and quantified analysis (bottom). Values represent three independent experiments. **d** Cell apoptosis was measured by flow cytometry analysis following Annexin V and PI staining. Experiment was repeated three times in triplicate. Images shows a representative result (top). Q3-1 necrosis cells. Q3-2 late apoptosis cells. Q3-3 normal cells. Q3-4 early apoptosis cells. Graphs (bottom) show quantitative analysis of early apoptosis, late apoptosis and total apoptosis (*P < 0.05, **P < 0.01, ***P < 0.001)

Plasmids carrying three different NRBP1 shRNAs were generated to knockdown NRBP1 expression in RKO and DLD1 cells (shRNA-1, shRNA-2, shRNA-3). A reduced protein level of NRBP1 in RKO and DLD1 cells transduced with shRNA-2 and shRNA-3 was verified by western blot (Fig. 4a). Cells transduced with shRNA-2 and shRNA-3 were retrieved for further analysis. Knockdown of NRBP1 by shRNR2 or shRNA-3 significantly enhanced proliferation ability in RKO and DLD1 cells (Fig. 4b, c). Markedly decreased apoptosis was observed in both cells transduced with shRNA compared to negative control cells (Fig. 4d). Taken together, these data suggest that NRBP1 possesses growth inhibitory and apoptosis enhancing activities and indeed acts as a tumour suppressor.

### NRBP1 regulates caspase-dependent intrinsic apoptosis in CRC cells

**Fig. 5:**
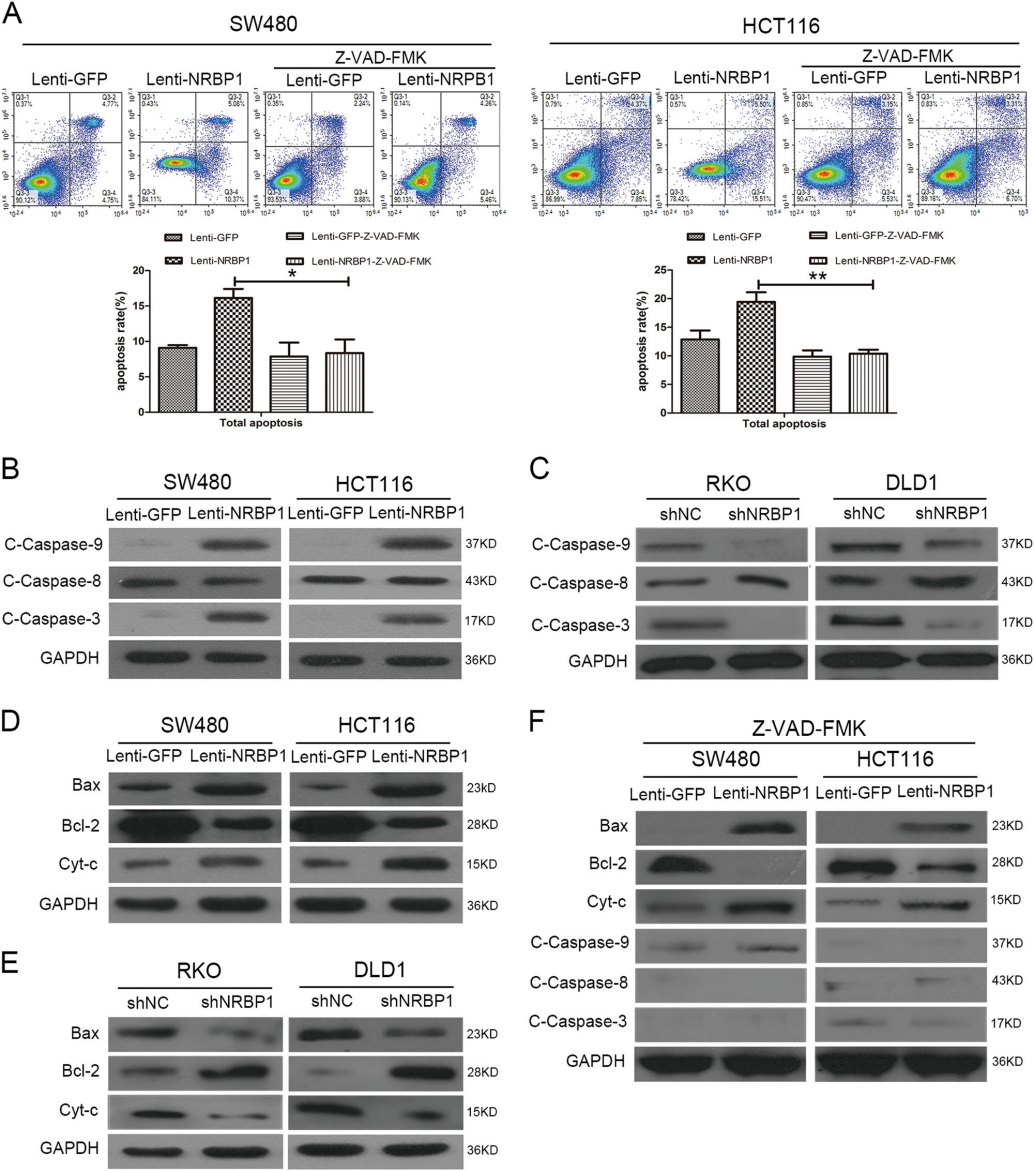
NRBP1 regulates caspase-dependent intrinsic apoptosis in CRC cells. **a** SW480 and HCT116 cells were treated with Z-VAD-FMK for 1 h, a pan-caspase inhibitor. Twenty-four hours later, apoptotic ratios were analysed by double staining the cells with Annexin V and PI using flow cytometry. Representative images from triplicate experiments are shown (top). Q3-1 necrosis cells. Q3-2 late apoptosis cells. Q3-3 normal cells. Q3-4 early apoptosis cells. Graphs (bottom) show quantitative analysis of total apoptosis. **b** Western blot was performed to investigate the expression levels of caspase-related proteins (caspase-9, caspase-8, caspase-3) in SW480 and HCT116 cells transduced with lenti-NRBP1 (lenti-GFP was used as a negative control). GAPDH was used as loading control. **c** Western blot was performed to investigate the expression levels of caspase-related proteins (caspase-9, caspase-8, caspase-3) in RKO and DLD1 cells transduced with shNRBP1. GAPDH was used as loading control. **d** Bax, Bcl-2, cytosolic cytochrome *c* and GAPDH (loading control) protein levels were detected by western blot in SW480 and HCT116 cells transduced with lenti-NRBP1 (lenti-GFP was used as a negative control). **e** Bax, Bcl-2, cytosolic cytochrome *c* and GAPDH (loading control) protein levels were detected by western blot in RKO and DLD1 cells transduced with shNRBP1. **f** The effects of Z-VAD-FMK on caspase-related protein in SW480 and HCT116 cells transduced with lenti-NRBP1 or lenti-GFPS were evaluated by western blot analysis (*P < 0.05, **P < 0.01)

As the caspase cascade has been known to have a key role in the execution of apoptosis, we examined whether enhanced apoptosis triggered by overexpression of NRBP1 in CRC cells was associated with the activation of caspases. CRC cells were treated with Z-VAD-FMK, a pan-caspase inhibitor. In the presence of Z-VAD-FMK, lenti-NRBP1 infection did not increase the percentage of Annexin V/PI positive cells in SW480 and HCT116 cells (Fig. 5a), indicating a critical role of caspases in the induction of apoptosis by NRBP1.

To confirm the suggestion that NRBP1 induces caspase-dependent cell death, the expression levels of the active form of caspases were detected. As shown in Fig. 5b and c, overexpression of NRBP1 increased the expression levels of cleaved caspase-9 and cleaved caspase-3 proteins (Fig. 5b), whereas silencing NRBP1 decreased the expression levels of cleaved caspase-9 and cleaved caspase-3 proteins detected using western blot (Fig. 5c). However, no alteration of the expression of cleaved caspase-8 was observed. Caspase-9 and caspase-8 are essential for the intrinsic and extrinsic apoptotic pathways, respectively. The active form of both directly cleaved downstream effector caspases, such as caspase-3, leads to the propagation of apoptosis. The results indicate that NRBP1 promotes apoptosis in CRC cells through intrinsic pathway with the activation of the caspase cascade.

Mitochondrial damage has a central role in intrinsic apoptosis, followed by cytochrome *c* release from mitochondria into the cytoplasm. The latter triggers the proteolytic activity of caspase-9 and caspase-3^14^. As shown in Fig. 5d, cytosolic cytochrome *c* levels increased in SW480 and HCT116 cells transduced with lenti-NRBP1 compared with control cells. Furthermore, cytosolic cytochrome *c* levels decreased in RKO and DLD1 cells transduced with shRNA compared to controls (Fig. 5e). Mitochondrial dysfunction is mediated by Bcl-2 proteins, which include both pro-apoptotic and anti-apoptotic members. The ratio between pro-apoptotic and anti-apoptotic proteins determines cell survival or death^15^. Western blot analysis showed that overexpression of NRBP1 in SW480 and HCT116 cells resulted in significantly increased expression of the pro-apoptotic protein Bax and significantly decreased expression of the anti-apoptotic protein Bcl-2 (Fig. 5d). As expected, knockdown of NRBP1 resulted in decreased expression of Bax and increased expression of Bcl-2 in DLD1 and RKO cells (Fig. 5e). An increased Bax/Bcl-2 protein ratio initiates the intrinsic pathway.

When cells were treated with Z-VAD-FMK, western blot results showed that although upregulation of Bax, downregulation of Bcl-2 and upregulation of cytosolic cytochrome *c* was not reversed, Z-VAD-FMK significantly eliminated the upregulation of cleaved caspase-9 and cleaved caspase-3 induced by overexpression of NRBP1 in SW480 and HCT116 cells (Fig. 5f). Taken together, these results suggest that the apoptosis induced by NRBP1 is caspase-dependent.

### NRBP1 promotes apoptosis through the activation of JNK in CRC cells

**Fig. 6:**
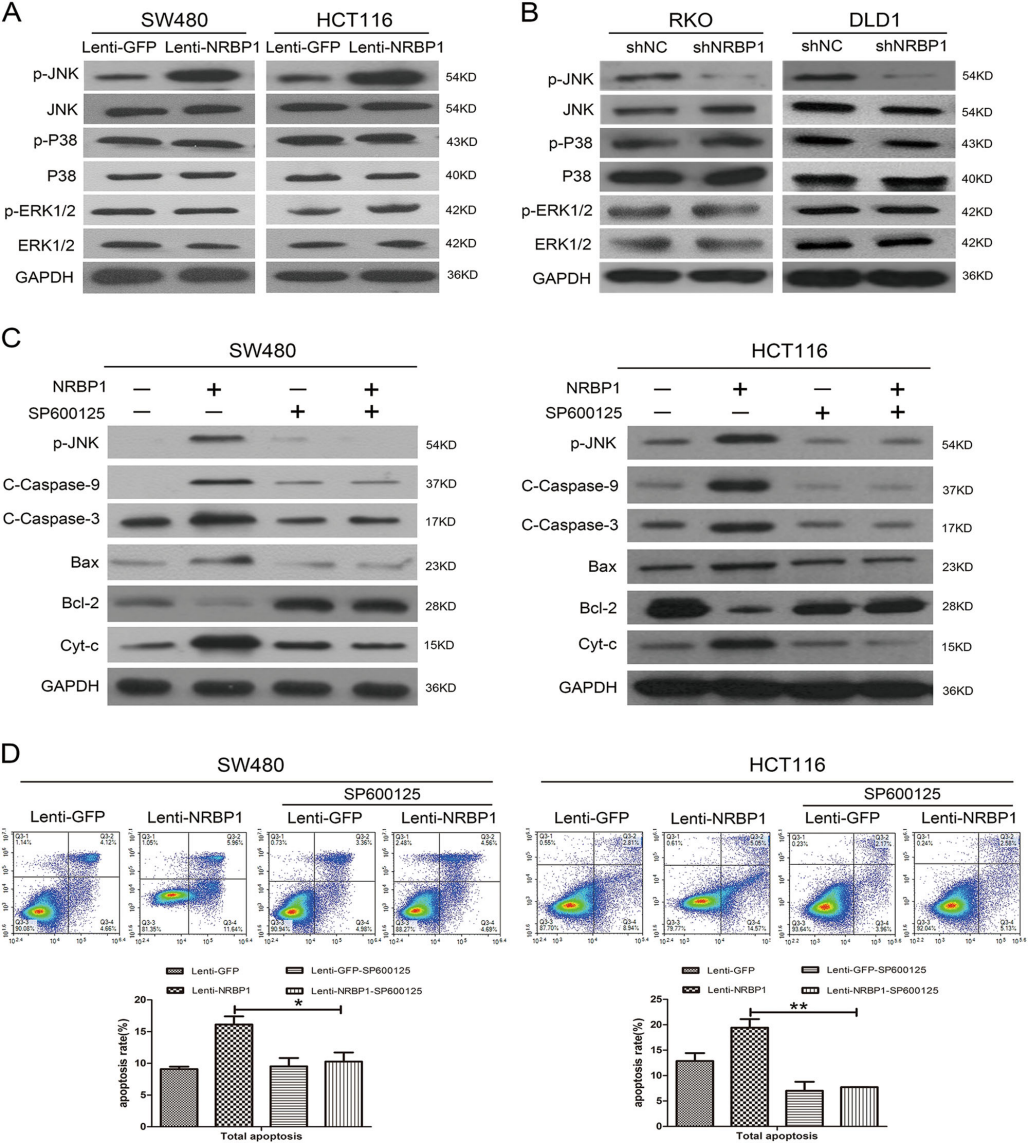
NRBP1 promotes apoptosis through the activation of JNK in CRC cells. **a** The effects of NRBP1 overexpression on targets in the JNK, P38, ERK1/2 signalling pathways were assessed by western blot in SW480 and HCT116 cells. GAPDH was used as loading control. **b** The effects of NRBP1 silencing on targets in the JNK, P38, ERK1/2 signalling pathways were assessed by western blot in RKO and DLD1 cells. GAPDH was used as loading control. **c** SW480 and HCT116 cells were treated with SP600125 for 30 min. Twenty-four hours later, caspase-related proteins were evaluated by western blot. **c** Apoptotic ratios were analysed by double staining the cells with Annexin V and PI using flow cytometry. Representative images from triplicate experiments are shown (top). Q3-1 necrosis cells. Q3-2 late apoptosis cells. Q3-3 normal cells. Q3-4 early apoptosis cells. Graphs (bottom) show quantitative analysis of total apoptosis (*P < 0.05, **P < 0.01)

To explore the underlying signal transduction pathways involved in the apoptosis process triggered by NRBP1 in CRC cells, the effect of NRBP1 on the actions of JNK, P38 and ERK1/2 MAPK was examined. As revealed by western blot, overexpression of NRBP1 enhanced the levels of phosphorylated JNK in SW480 and HCT116 cells without changes in total JNK protein levels. Moreover, no changes in the levels of phosphorylated and total P38 and ERK1/2 were observed (Fig. 6a). In contrast, reduced levels of phosphorylated JNK were detected in RKO and DLD1 cells transduced with shRNA (Fig. 6b).

To further confirm whether the activation of the JNK signalling pathway is involved in this apoptotic phenomenon, the JNK-specific inhibitor SP600125 was used. Cells were incubated with SP600125 in serum-free medium for 30 min. As shown in Fig. 6c, upregulation of phosphorylated JNK, cleaved caspase-9, cleaved caspase-3, Bax, and cytosolic cytochrome *c* and downregulation of Bcl-2 induced by NRBP1 overexpression were prevented by SP600125 in both SW480 and HCT116 cells. Furthermore, the JNK blocker significantly attenuated the proportion of NRBP1-induced apoptosis from 16.13 ± 1.27% to 10.27 ± 1.44% and from 19.43 ± 1.68% to 7.73 ± 0.02% in SW480 cells and HCT116 cells, respectively. (Fig. 6d). These data suggest that the activation of JNK signalling is involved in the positive regulation of NRBP1 in CRC cell apoptosis.

### Inhibition of tumour growth by NRBP1 in vivo

**Fig. 7:**
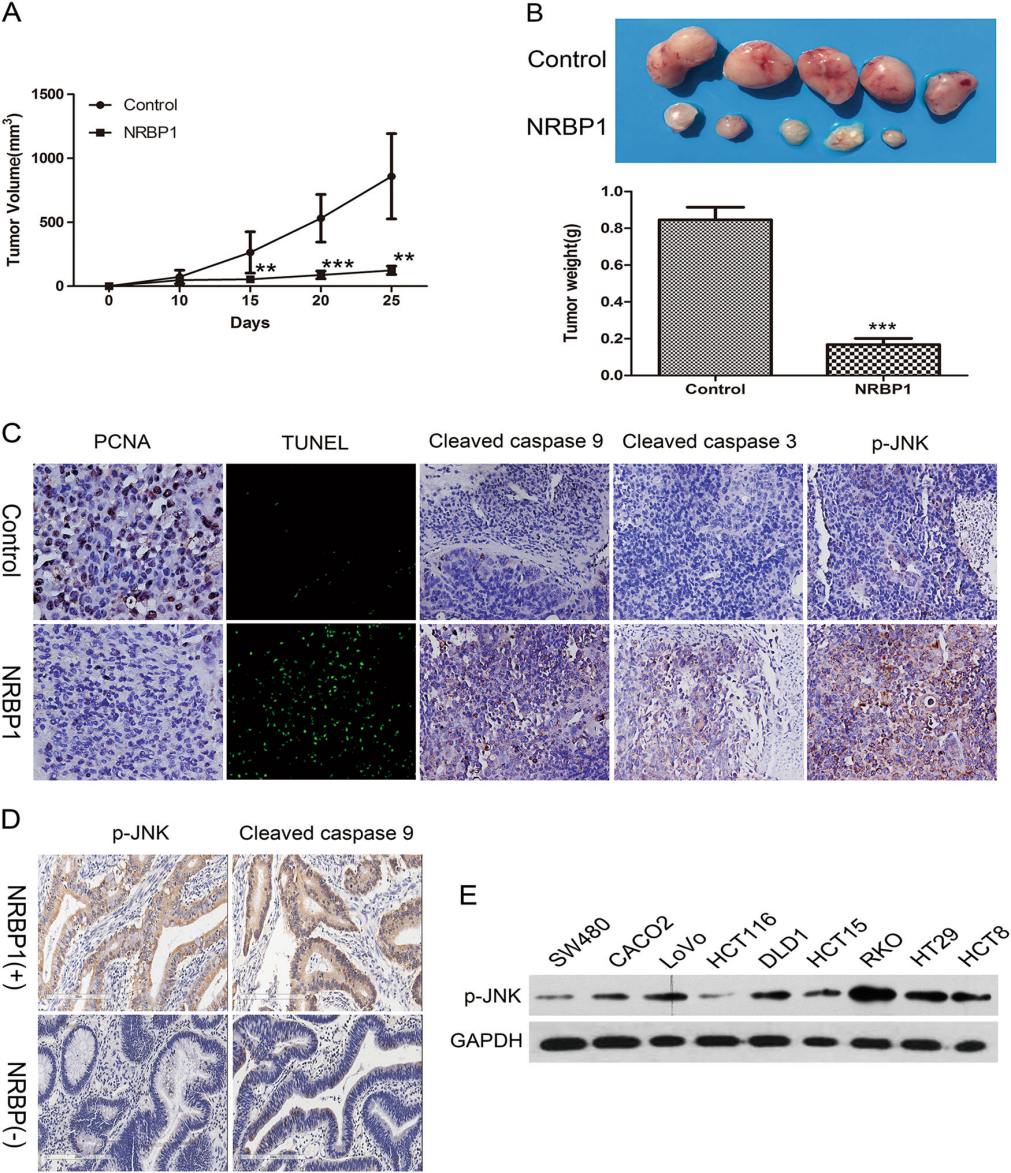
Inhibition of tumour growth by NRBP1 in vivo, and association of NRBP1 and apoptosis-related proteins in CRC tissue specimens and cell lines. **a** Female nude were subcutaneously injected with SW480-NRBP1 and SW480-GFP cells. Tumours were measured using digital calipers every 5 days. Tumour volume was calculated and recorded. The mean volumes in mice injected with SW480-NRBP1 cells were significantly smaller than in the controls. **b** On day 25 after injection, mice were killed and the tumours were removed and weighed. Image shows the photograph of representative xenograft tumours of two different treatment groups. **c** Expression of PCAN, cleaved caspase-9, cleaved caspase-3 and phosphorylated JNK was determined by IHC staining in tumour xenografts. Cell apoptosis was analysed by TUNEL staining. **d** Expression of phosphorylated JNK and cleaved caspase-9 was determined by IHC staining in CRC tissues. **e** The expression of phosphorylated JNK in the nine human CRC cells was assayed by western blot. The expression of GAPDH was used as loading control. (**P < 0.01, ***P < 0.001)

A xenograft mouse model was established to investigate the influence of NRBP1 on tumour growth in vivo. SW480-NRBP1 and SW480-GFP (control) cells were injected subcutaneously into nude mice, and tumour volume and tumour weight were measured. Tumours grew faster in the control group, and the mean volume of tumours derived from the cells transduced with lenti-NRBP1 was significantly smaller than those derived from cells transduced with lenti-GFP during the entire period (Fig. 7a). Moreover, on day 25 after injection, when the tumours were harvested, the average weight of tumours was significantly smaller in nude mice injected with SW480-NRBP1 cells relative to mice injected with empty vector cells (Fig. 7b). These findings in vivo were consistent with the results of the cell proliferation assay in vitro, suggesting that NRBP1 acts as a tumour suppressor in CRC carcinogenesis in vivo.

To further reveal the mechanism of NRBP1 suppression, IHC analysis and in situ TUNEL staining of tumour sections were performed. A reduction in tumour growth was accompanied by a decrease in proliferation (PCNA staining) and an increase in cell death (TUNEL staining). Increased expression levels of cleaved caspase-9, cleaved caspase-3 and phosphorylated JNK were observed in tumours derived from SW480-NRBP1 cells compared with tumours derived from SW480-GFP cells (Fig. 7c). These results confirm that NRBP1 promotes CRC cell apoptosis through inducing intrinsic apoptosis pathway mediated by JNK.

### Association of NRBP1 and apoptosis-related proteins in CRC tissue specimens and cell lines

The expression levels of cleaved caspase-9 and phosphorylated JNK were assessed using IHC in the above 30 pairs of CRC tissues and their corresponding normal colorectal mucosal tissues. Among cancer tissues, negative or low expression of phosphorylated JNK protein was detected in 13 of 30 samples, and high expression levels were detected in 17 of 30 samples. To address the relationship between NRBP1 and phosphorylated JNK in this set of CRC samples, co-expression of these two proteins in the same specimens was analysed. Twelve samples simultaneously expressed high levels of NRBP1 and phosphorylated JNK, while 10 samples simultaneously showed low expression. We found a statistically significant correlation between NRBP1 and phosphorylated JNK expression in CRC samples (*P* = 0.009, *r* = 0.471, Fig. 7d; Table 5). Interestingly, we also found a strong correlation between the expression of NRBP1 and phosphorylated JNK in human CRC cells (Fig. 7e).

**Table 5:** Association between NRBP1 and p-JNK, C-Caspase-9 expression in CRC tissues

Variable	NRBP1, *n* (%)		*r*	*P*
	Low	High		
p-JNK				
Low or negative	10	3	0.471	0.009
High	5	12		
C-Caspase-9				
Low or negative	12	2	0.668	<0.001
High	3	13		

In addition, Spearman rank correlation analysis showed that low NRBP1 expression was significantly correlated with low expression of cleaved caspase-9 (*P* < 0.001, *r* = 0.668, Fig. 7d; Table 5).

## Discussion

NRBP1 is a ubiquitously expressed and highly conserved pseudokinase, which has important roles in cellular homoeostasis as it binds with a number of key transcription factors and ubiquitination machinery. However, NRBP1 has only recently been described to have an important role in cancer^16^. Wilson et al.^11^ reported downregulated expression of NRBP1 in a range of cancers. However, Ruiz et al.^13^ found that the expression of NRBP1 was mostly restricted to high-grade prostate intraepithelial neoplasia and prostate cancer samples but absent in normal prostate specimens. In the present study, we observed that NRBP1 expression levels were significantly reduced in CRC tissues compared with corresponding adjacent normal tissues, which is consistent with the findings of Wilson et al.^11^ concerning CRC, suggesting that NRBP1 functions as a tumour-suppressor gene in CRC. A previous study^12^ showed that overexpression of NRBP1 inhibited breast cancer cell proliferation. In our study, we found that NRBP1 not only affected CRC cell proliferation ability but also influenced apoptosis of CRC cells. Apoptotic cell ratios increased in NRBP1 overexpression cells, whereas the percentage of apoptotic cells significantly decreased in NRBP1 knockdown cells. Thus, the results indicate that NRBP1 inhibits tumour growth by promoting the apoptosis of CRC cells.

Next, we investigated the clinical implications of NRBP1 in CRC to further verify the suppression role in CRC. Although a strong correlation was reported between NRBP1 expression and histopathology grades, TNM stage, tumour diameter and lymph node involvement in breast cancer^12^, we found a significant correlation only between NRBP1 expression level and TNM stage; early TNM stage was frequently associated with high NRBP1 expression. Previous studies have reported the prognostic value of NRBP1 expression in cancer. In lung cancer and breast cancer, low NRBP1 is significantly correlated with poor prognosis^11,12^. In contrast, in prostate cancer, high NRBP1 expression was linked with poor clinical outcome^13^. In our current study, we demonstrated that patients with high NRBP1 expression had a longer OS and DFS than those with low NRBP1 expression in CRC, and the Cox regression model showed that a high NRBP1 level was an independent indicator of favourable prognosis.

The caspase protein family has a key role in the initiation and execution of apoptosis^17^. There are two main signalling pathways involved in apoptosis: the extrinsic or death receptor pathway and the intrinsic or mitochondrial pathway. Activation of caspase-8 is critical for the extrinsic pathway, whereas the intrinsic pathway requires the activation of caspase-9. Both caspase-9 and caspase-8 activate caspase-3, leading to cellular death^18^. Here, we have shown that enhanced apoptosis mediated by overexpression of NRBP1 was attenuated by treatment with a general caspase inhibitor, Z-VAD-FMK, indicating that NRBP1 promotes the apoptosis of CRC cells in a caspase-dependent manner. Moreover, we found that the apoptosis induced by NRBP1 in CRC cells occurred only via the intrinsic pathway, as evidenced by increased cleaved caspase-9 and cleaved caspase-3 in NRBP1-overexpressing CRC cells and decreased cleaved caspase-9 and cleaved caspase-3 in NRBP1-silenced CRC cells; upregulation of cleaved caspase-9 and cleaved caspase-3 caused by NRBP1 overexpression was abolished by the caspase inhibitor. The intrinsic pathway is regulated mainly by the Bcl-2 protein family, which includes pro-apoptotic members and anti-apoptotic members. Upregulation of pro-apoptotic proteins and downregulation of anti-apoptotic proteins induce mitochondrial outer membrane permeabilization, a key event in the initiation of apoptosis, resulting in cytochrome *c* release from the mitochondria into the cytosol and leading to caspase activation^19–21^. We showed that overexpression of NRBP1 significantly increased the expression of the pro-apoptotic protein Bax and decreased the expression of the anti-apoptotic protein Bcl-2; moreover, cytosolic cytochrome *c* was also elevated in NRBP1-overexpressing cells compared to control cells. On the other hand, knockdown of NRBP1 decreased the expression of Bax and cytosolic cytochrome *c* and increased the expression of Bcl-2. These results collectively confirm that NRBP1 is involved in CRC cell apoptosis via the intrinsic pathway.

Because NRBP1-regulated CRC cell apoptosis is caspase-dependent, the next pertinent question that needs to be answered is through which signal NRBP1 activates intrinsic apoptosis. JNK, a master protein kinase and a key member of the MAPKs, has an important role in a variety of physiological and pathological processes, including cell proliferation, differentiation, survival and death^22^. The diversity of cellular functions of JNK determines the diversity of the role of JNK in different disease conditions, including cancer. Activation of the JNK pathway either contributes to tumour development or suppresses tumour development resulting from its well-recognised apoptotic functions depending on the tissue or cell type^23,24^. In our study, we provide conclusive evidence of the involvement of JNK in the onset of the intrinsic apoptotic pathway triggered by NRBP1 in CRC cells. First, we showed that elevated levels of phosphorylated JNK were accompanied by pro-apoptotic protein upregulation, anti-apoptotic protein downregulation, and upregulation of cytosolic cytochrome *c*, cleaved caspase-9 and cleaved caspase-3 in NRBP1-overexpressing SW480 and HCT116 cells. Second, a significant positive correlation between NRBP1 expression and phosphorylated JNK, cleaved caspase-9 expression was observed in CRC tissue samples and in the xenograft model. Previous studies have reported that JNK regulates apoptosis through two distinct pathways: on one hand, active JNK activates AP-1 and increases the expression of Fas/FasL signalling pathway-related proteins. Binding of FasL to Fas further triggers extrinsic apoptosis^25^. On the other hand, JNK inactivates anti-apoptotic protein Bcl-2 to cause the permeabilization of the mitochondrial membrane, followed by the release of cytochrome *c* and the activation of caspase-9 and caspase-3, which is in accordance with our results^26^. Moreover, our results suggest that the overexpression of NRBP1 induced the activation of JNK to not only decrease Bcl-2 expression but also increase the level of Bax in CRC cells. Bax may translocate to the mitochondria, leading to the permeabilization of the mitochondrial membrane^27^. Third, when JNK was blocked by SP600125, we found that upregulation of phosphorylated JNK, Bax, cytosolic cytochrome *c*, cleaved caspase-9, and cleaved caspase-3 and downregulation of Bcl-2 induced by NRBP1 overexpression were prevented. Moreover, enhanced apoptosis induced by overexpression of NRBP1 was attenuated by the JNK blocker. On the basis of these data, we determined that the activation of JNK has a role in NRBP1-induced apoptosis in CRC.

JNK is activated by upstream MAP2Ks (MKK4 and MKK7 kinases), which directly phosphorylate the Thr-Pro-Tyr motif. MAP2Ks are activated via phosphorylation on two serine residues by MAP3Ks, and in turn, MAP3Ks are activated through mechanisms mediated by GTPases, including rat sarcoma (RAS), Ras-related C3 botulinum toxin substrate (RAC), cell division cycle 42 (CDC42) and Ras homologous (Rho) in response to multiple stimuli. It has been previously reported that NRBP1 binds RAC to form a complex. Whether the RAC-NRBP1 complex is involved in regulating JNK requires further analysis.

In summary, our observations have for the first time found a novel positive correlation between NRBP1 and intrinsic apoptosis, which is mediated by the activation of JNK. Overexpression of NRBP1 triggers the activation of caspase-dependent intrinsic apoptosis, which promotes CRC cell apoptosis and inhibits cell proliferation and colony formation. High NRBP1 expression levels are associated with longer OS and DFS. Our study indicates a role of NRBP1 in suppressing CRC, and the NRBP1/JNK/intrinsic apoptosis axis could be a potential target for CRC treatment. However, further study is needed to clarify the specific molecular mechanisms by which NRBP1 impinges on the JNK-mediated apoptotic pathway.

## Materials and methods

### Patients and tissue specimens

This study was approved by the institutional review boards of Sun Yat-Sen University (Guangzhou, China). All patients provided written informed consent. Thirty paired freshly frozen and thirty paired formalin-fixed, paraffin-embedded (FFPE) cancer issues and their matched normal tissues were obtained from CRC patients who underwent surgery at the Sixth Affiliated Hospital of Sun Yat-sen University. These samples were used for qRT-PCR and IHC analysis.

Paraffin-embedded CRC specimens from 360 patients who received initial surgical resection of the lesions in the First Affiliated Hospital of Sun Yat-sen University and the Sixth Affiliated Hospital of Sun Yat-sen University from January 2004 to December 2008 were retrieved for tissue microarray analysis. The histological status of all patients was independently confirmed by two pathologists. None of the patients received chemotherapy or radiotherapy prior to surgery. Information on clinicopathological features was collected from clinical records and pathological reports.

### Cell lines and reagents

CRC cell lines were purchased from the American Type Culture Collection (ATCC, Manassas, VA). These cells were maintained according to instructions described by ATCC. Z-VAD-FMK and SP600125 were purchased from Sigma-Aldrich (St. Louis, MO, USA).

### RNA extraction and qRT-PCR

Total RNA was extracted from CRC tissue cells using Trizol reagent (Invitrogen) as recommended by the manufacturer’s protocol. Reverse transcription of RNA into cDNA primed with an oligo (dT) primer was carried out using the First-Strand Synthesis System (Fermentas, Burlington, Ontario). Using SYBR Green (Toyobo, Osaka, Japan) as a double-stranded DNA-specific fluorescent dye, real-time PCR analysis was carried out to quantify mRNA expression on the ABI 7500HT system (Applied Biosystems, Foster City, CA, USA). The primer sequences for NRBP1 and β-actin are listed as follows:

NRBP1 forward: 5′-GAGGTGAATCAACGGAATGTACC-3′; reverse: 5′-CTTGTAGTTCTTGCGTTCAGAGA-3′.

β-actin: forward: 5′-TCCTGACCCTGAAGTACCCCATTG-3′; reverse: 5′-GGAACCGCTCATTGCCGATAGT-3′.

The expression levels of mRNA from all samples were normalised to the internal gene β-actin, and fold changes were determined by the comparative Ct method^28^.

### TMAs

Three hundred sixty donor tissues that were formalin-fixed and paraffin-embedded and their corresponding haematoxylin and eosin-stained slides were carefully selected from the archives. All cases of haematoxylin and eosin-stained slides were reviewed so that the diagnosis of carcinoma could be confirmed, and suitable donor blocks could be selected. Two cylindrical cores of 1 mm diameter from the selected paraffin wax block were retrieved and transferred to recipient TMA blocks. We constructed TMAs through an automated TMA instrument (ALPHELYS, Plaisir, France).

### IHC staining

Sections were deparaffinized, heated at 100 °C in citrate buffer (10 mM, pH 6) to retrieve antigen and incubated with 3% H_2_O_2_ to block endogenous peroxidase activity. Primary antibody against NRBP1 (1: 100, Abnova), cleaved caspase-9 (1:200, Cell Signaling, Beverly, MA, USA), cleaved caspase-3 (1:200, Cell Signaling), phospho-JNK (1:100, Cell Signaling) and PCNA (1:8000, Cell Signaling) was applied, and the tissue sections were retained overnight at 4 °C. Next, slides were incubated with secondary antibody (Dako, EnvisionSystem/DAB-chromogen, Glostrup, Denmark), followed by staining with diaminobenzidine and counterstaining with haematoxylin.

To validate the specificity of the NRBP1 antibody, sections from stomach tissue with robust detectable NRBP1 protein and sections from thymus with low NRBP1 expression^7^ were subjected to IHC. The negative control sections were incubated with a normal mouse IgG to replace the primary antibody as recommended by Hewitt et al.^29^

Two pathologists blinded to clinicopathological data independently reviewed and scored immunostaining specimens on the TMAs. NRBP1 expression was scored based on the extent and intensity of staining. The extent was defined according to the percentage of positively stained tumour cells: 0–25% (1), 26–50% (2), 51–75% (3) and 76–100% (4). The intensity of staining was graded as weak (1), moderate (2), or strong (3). The final expression score of each case was calculated by multiplying the extent and intensity score.

A logical cut-off score was generated by ROC curve so that the expression of NRBP1 could be dichotomised to facilitate further survival analysis. For each IHC score, the sensitivity and specificity for OS and DFS of the study plotted to yield ROC curves. We selected the points localised at both maximum sensitivity and specificity as the cut-offs^30^. Specifically, the NRBP1 IHC cut-off scores for OS and DFS were 6.5 and 6.5, respectively. Therefore, we selected 6.5 as the cut-off for dividing CRC tissues into high and low expression levels.

### Western blot

We extracted proteins from cells using the Total Protein Kit (KeyGen, Nanjing, China) according to the manufacturer’s instructions; moreover, to detect the cellular localisation of cytochrome *c*, nuclear and cytoplasmic fractions were isolated using the Nuclear and Cytoplasmic Protein Extraction kit (KeyGen) according to the manufacturer’s instructions. Then, we separated the proteins using SDS-PAGE. After transferring protein to a polyvinylidene fluoride membrane (Pall, New York, USA), the membrane was blocked in 5% non-fat milk and then incubated with mouse anti-NRBP1 antibody (1:1000, Abnova) and mouse anti-cleaved caspase-9 antibody (Cell Signalling), mouse anti-cleaved caspase-3 antibody (Cell Signalling), mouse anti-cleaved caspase-8 antibody (Cell signalling), rabbit anti-Bax antibody (Cell signalling), mouth anti-Bcl-2 antibody (Cell signalling), rabbit anti-cytochrome *c* antibody (Cell signalling), rabbit anti JNK antibody (Cell signalling), rabbit anti-phospho-p38 antibody (Cell Signaling), rabbit anti-p38 antibody (Cell Signaling), rabbit anti-phospho-ERK1/2 antibody (Cell Signaling), rabbit anti-ERK1/2 antibody (Cell Signaling) or rabbit anti-GAPDH (Cell Signaling) at 1:1000 dilutions overnight at 4 °C, followed by HRP-linked secondary antibody (Cell Signaling) for 1 h at room temperature. The membrane was visualised using the ECL kit (Pierce Chemical Co, Rockford, IL). The assays were performed for three times. The density of each band was quantified by scanning densitometry and was analysed by Image J software (NIH, Bethesda, USA), and all protein expression levels were evaluated relative to GAPDH expression.

### Lentivirus infections

We purchased a lentivirus encoding the human NRBP1 gene and green fluorescent proteins (GFP) from Shanghai GenePharma Co Ltd, China. The target cells were infected with the lentivirus according to the manufacturers’ instructions. A fluorescence microscope camera was used to observe the transfection effect 72 h after infection, and NBRP1 expression was confirmed by western blot.

### RNA interference

Three pairs of short interfering RNA (siRNA) targeting the NRBP1 sequence (GGCCATGGATACAGAGGAA, CCTTGAAGATGTCAGGAAT, GTCGAGAAGAGCAGAAGAA) were synthesised. Oligonucleotides representing the siRNA targeting NRBP1 or scramble siRNA (negative control, NC) were cloned into the pSliencer4.1-CMV neo vector (Ambion) between the *Bam*HI and *Hin*dIII sites following the manufacturer’s instructions. Cells were transfected with shRNA for knockdown of endogenous NRBP1 using Lipofectamine 3000 transfection reagent (Invitrogen). Total protein was collected from cells to analyse the suppression efficiency.

### Cell proliferation assay

Cell proliferation was measured via the Cell Counting Kit-8 (CCK-8) (Dojindo, Kumamoto, Japan) assay according to the manufacturer’s instructions. After transfection with lenti-NRPB1 or shRNA and their corresponding control, 100 μL of cells were seeded into 96-well plates at a concentration of 2000 cells per well and cultured. At 0, 24, 48 and 72 h, cells were stained with 10 μL CCK-8 reagent and incubated at 37 °C for 2 h. Finally, the number of viable cells was assessed by measuring the absorbance at a wavelength of 450 nm in each well.

### Colony formation assay

For the colony formation assay, transfected cells were seeded in 6-well plates with G418 at 3000 cells per well. Cultures were maintained for fourteen to twenty-one days. At the end of the experiment, cells were fixed with 70% ethanol and stained with 0.5% crystal violet (Sigma-Aldrich) for 30 min. Colonies with 50 cells or more were counted.

### Flow cytometry analysis

Apoptosis was determined with the Annexin V-FITC/propidium iodide (PI) double staining kit (BD Biosciences, San Diego, CA, USA). After 72 h of transfection, cells were harvested and fixed in 70% cold ethanol at -20 °C overnight, followed by incubation with FITC-labelled Annexin V and PI for 30 min in the dark. The extent of cell apoptosis, indicated as the percentage of apoptotic cells, was measured using a FACSCalibur flow cytometer (BD Biosciences, Franklin Lakes, NJ, USA). All experiments were performed three times.

### Animal model and histological analysis

Four- to six-week-old female nude (BALB/C nu/nu) mice, purchased from Sun Yat-Sen University’s Laboratory Animal Canter (Guangzhou, China), were used to generate a subcutaneous xenograft mouse model. SW480 cells transduced with lenti-NRBP1 or lenti-GFP were harvested, and approximately 1 × 10^7^ cells in 200 μL PBS were injected subcutaneously into each mouse. Mice were monitored daily, and tumour growth was evaluated every five days by measuring the length and width of the tumour mass with callipers. The tumour volume was calculated by the following formula: (length × width^2^)/2. Twenty-five days after injection, the mice were killed, and the tumours were removed and weighed. The tumour tissues were fixed in 4% formalin and then embedded in paraffin for further IHC staining and TUNEL staining. TUNEL staining was conducted following the manufacturer’s instructions (Promega, Madison, WI, US). All animal procedures were conducted following the Guide for the Care and Use of Laboratory Animals and were approved by the Animal Experiment Administration Committee of the Medical School of Sun Yat-sen University.

### Statistical analysis

IBM SPSS Statistics 22 were employed for data analysis. The association between NRBP1 expression and clinicopathological parameters was assessed with Pearson *χ*^2^ tests. Survival curves were plotted using the Kaplan–Meier method and compared using the log-rank test. Univariate and multivariate analyses were performed by applying the Cox proportional hazards test. The differences between two groups were analysed using an independent *t*-test. The correlation between gene expression was analysed using Spearman’s test. A *P* value (two-sided) < 0.05 was considered to be significant.

## Electronic supplementary material

Supplementary Figure S1

Supplementary Figure S2

Supplementary Figure S3

Supplementary Figure Legends
