# Supplementary figures and images for: Nuclear receptor binding protein 1 correlates with better prognosis and induces caspase-dependent intrinsic apoptosis through the JNK signalling pathway in colorectal cancer
Source: Cell Death Dis. 2018 Mar 22;9(4):436. doi: 10.1038/s41419-018-0402-7 (PMC5864759; doi:10.1038/s41419-018-0402-7)

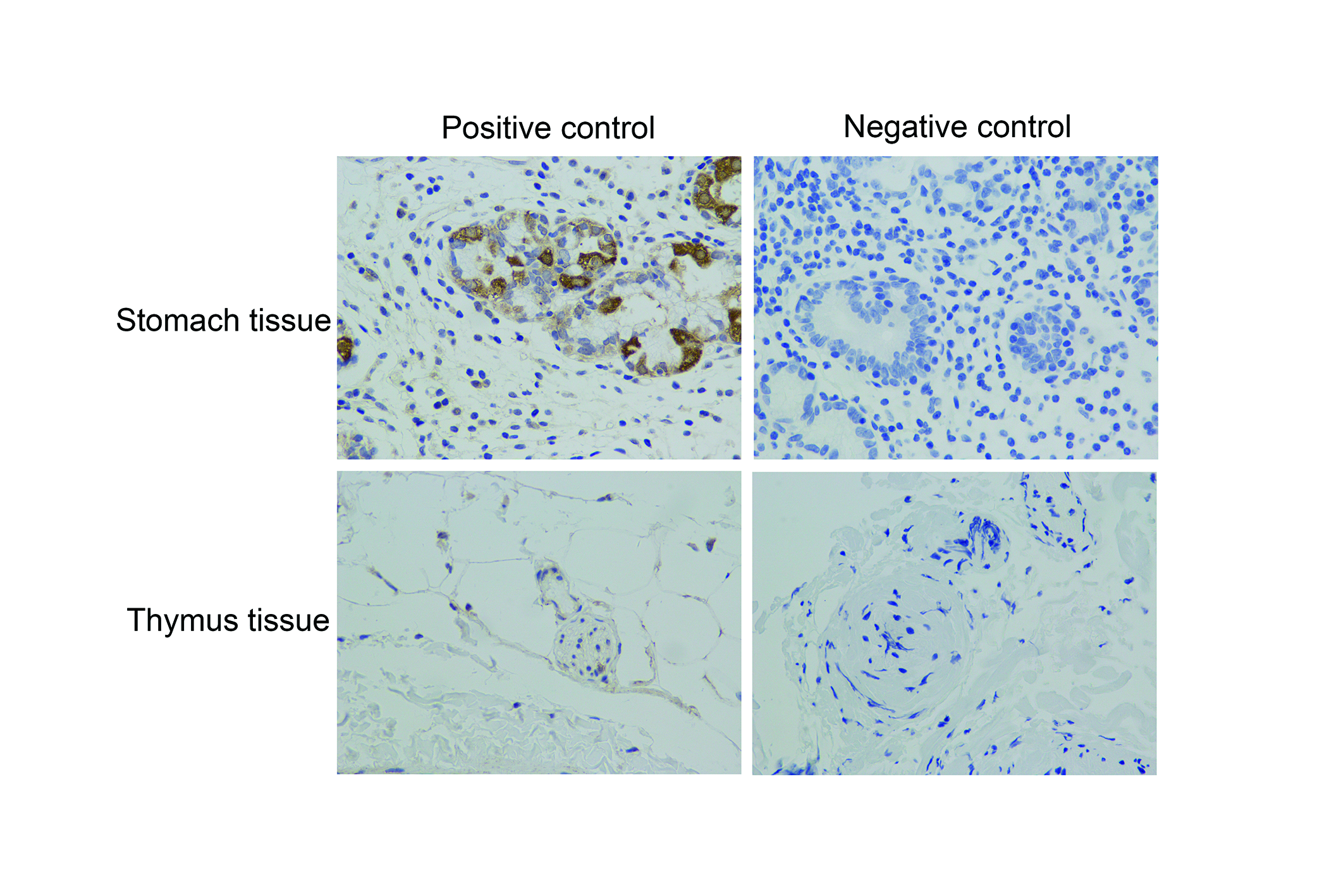

Supplement: Supplementary file 1 — Supplementary Figure S1 [file 41419_2018_402_MOESM1_ESM.tif]

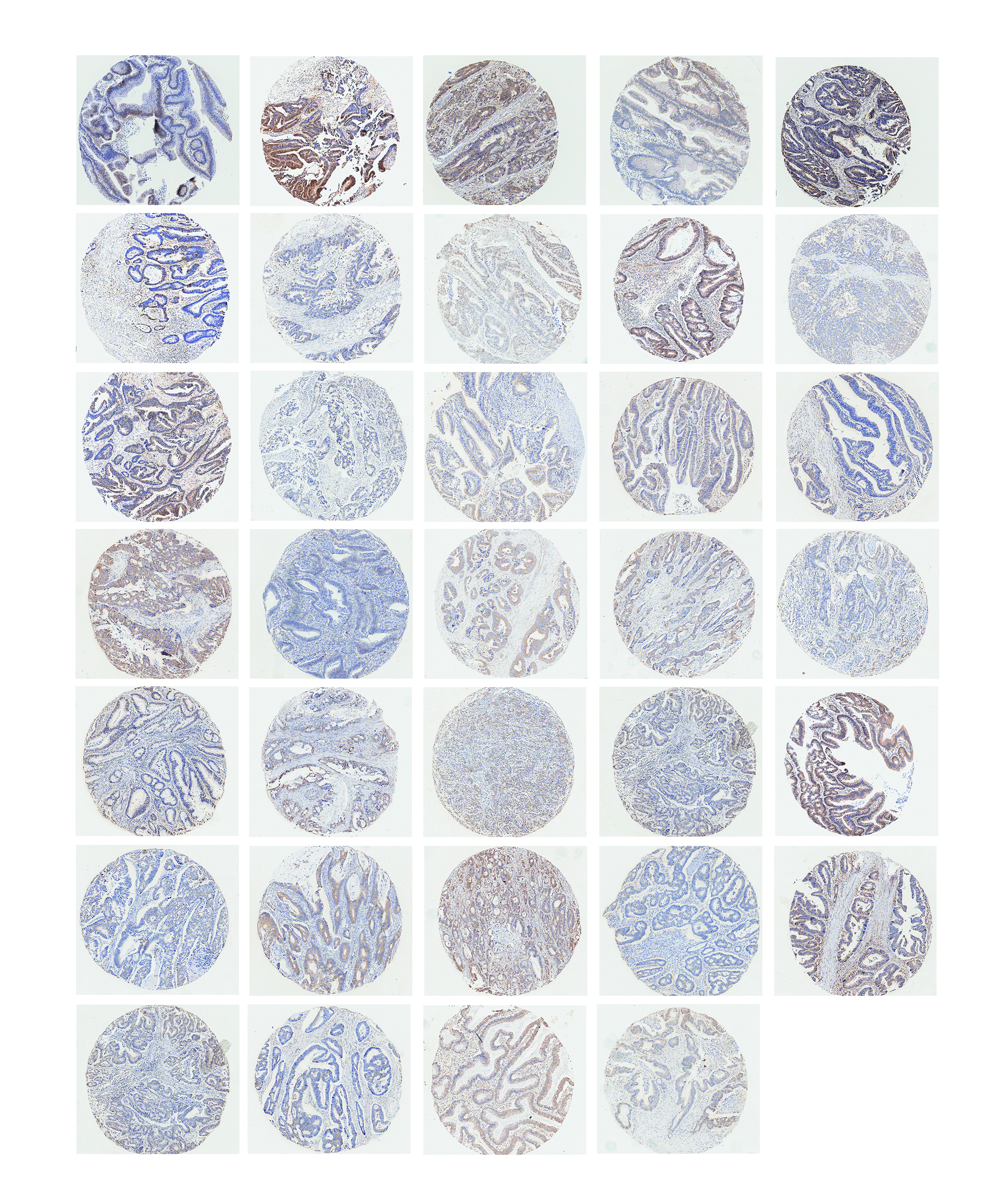

Supplement: Supplementary file 2 — Supplementary Figure S2 [file 41419_2018_402_MOESM2_ESM.tif]

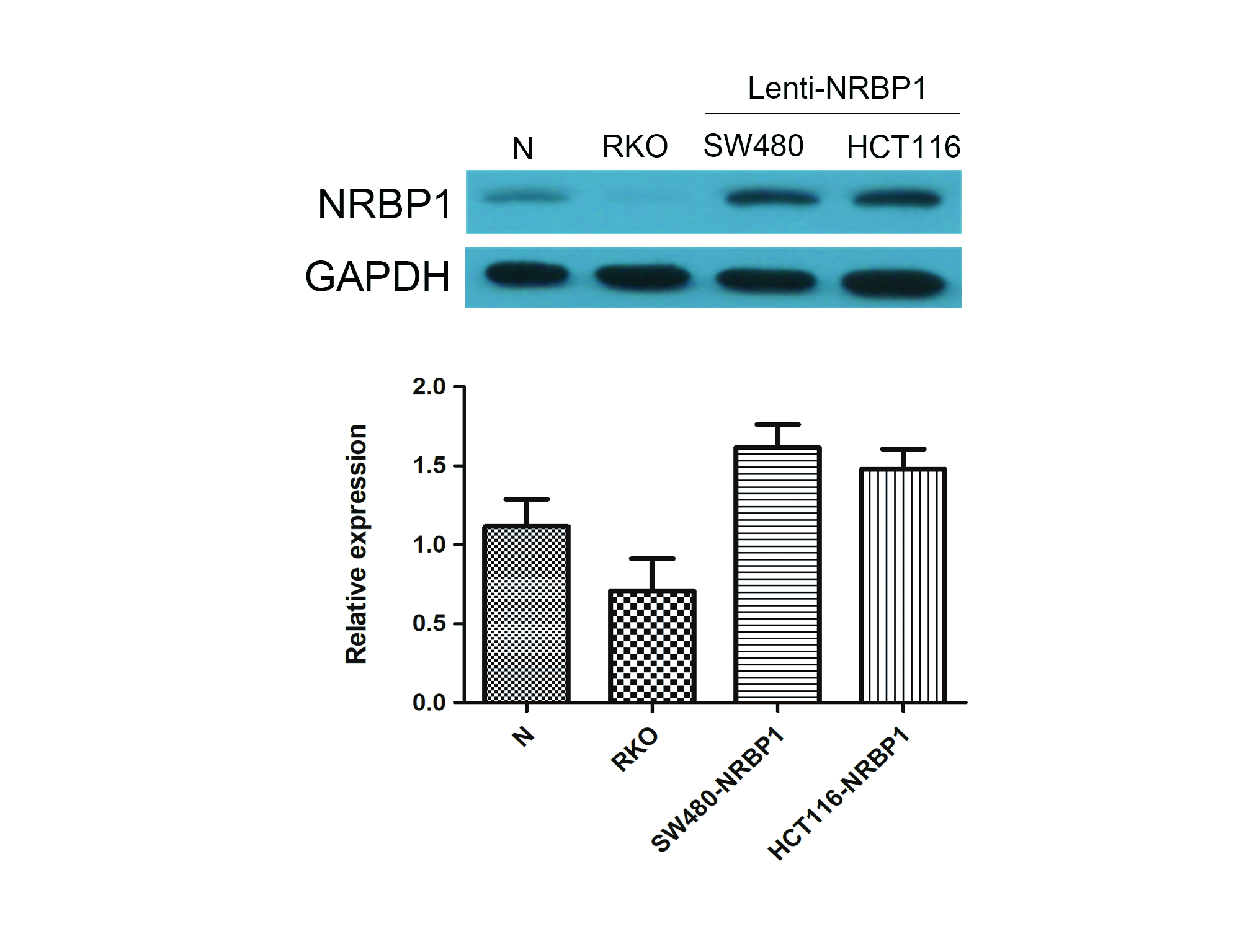

Supplement: Supplementary file 3 — Supplementary Figure S3 [file 41419_2018_402_MOESM3_ESM.tif]
