# Supplementary material for: Nuclear receptor binding protein 1 correlates with better prognosis and induces caspase-dependent intrinsic apoptosis through the JNK signalling pathway in colorectal cancer
Source: Cell Death Dis. 2018 Mar 22;9(4):436. doi: 10.1038/s41419-018-0402-7 (PMC5864759; doi:10.1038/s41419-018-0402-7)
Supplement: Supplementary file 4 — Supplementary Figure Legends [file 41419_2018_402_MOESM4_ESM.docx]

**Supplementary Figure Legends**

**Supplementary Figure S1** To validate the specificity of the NRBP1 antibody, sections from stomach tissue with robust detectable NRBP1 protein and sections from thymus with low NRBP1 expression were performed IHC. The positive control sections were incubated with NRBP1 antibody. The negative control sections were incubated with a normal mouse IgG to replace the primary antibody.

**Supplementary Figure S2** IHC analysis for 34 CRC samples.

**Supplementary Figure S3** The NRBP1 expression levels were evaluated by western blot in normal colon tissue, RKO cell and SW480 cell and HCT116 cell transduced with lenti-NRBP1.
